# Self-positioning in space science communication: A corpus-assisted discourse study

**DOI:** 10.1371/journal.pone.0353260

**Published:** 2026-07-09

**Authors:** Fangfang Chen, Cindy Sing Bik Ngai, Ming Liu

**Affiliations:** 1 Faculty of Humanities, The Hong Kong Polytechnic University, Hong Kong, China; 2 Department of Language Science and Technology, The Hong Kong Polytechnic University, Hong Kong, China; 3 Department of Language Science and Technology, The Hong Kong Polytechnic University, Hong Kong, China; Shandong University, CHINA

## Abstract

This study incorporates text mining into critical discourse analysis to examine how government science agencies in China and the United States position themselves in space science communication on social media. Two specialized corpora have been built by collecting posts from government science agencies on Weibo from China and X (formerly Twitter) from the U.S. With the help of the text mining tool KH Coder, this study gives a corpus-assisted discourse analysis of the particular ways of positioning at different levels of discourse: (1) topics/themes, (2) addressing terms, and (3) those words that co-occur with self-addressing terms in a sentence. The findings reveal significant differences in their preferential ways of positioning. Chinese government science agencies present themselves as state-affiliated yet approachable institutions, blending achievements and operational efficiency with patriotism and collective pride. Their use of diverse addressing terms and co-occurrence patterns portrays them as experienced, supportive guides, balancing national identity with interpersonal closeness. In contrast, U.S. government science agencies emphasize professionalism, focusing on research, space exploration, mission execution, and audience engagement, with minimal reference to state affiliation. Their addressing terms are formal and standardized, with pride centred on mission success and discovery, highlighting expertise and scientific leadership. Their preferential ways of positioning are further explained in their respective contexts in order to present a proper understanding of these differences.

## 1. Introduction

In recent years, space exploration has attracted increasing global attention and underscores expansion, marked by surging investments [1], a rapid rise in orbital asset launches [2], and the growing participation of private actors such as SpaceX [3]. The strategic importance of space exploration extends beyond scientific ambition; it enhances international prestige, boosts technological competitiveness, and also serves national security [4]. In this context, space science communication, which includes both the communication of space capability “development” and space “discovery”, strategically plays multiple social roles in its dialogue with the public [5]. It is not merely about conveying information; it is a discourse shaped by strategic intentions and sociopolitical implications.

Science communication is broadly defined as the use of knowledge-sharing methods to enhance public understanding, awareness and opinion formation around scientific topics [6]. Like other areas of science communication, such as climate and health communication, space science communication shares these core goals but also exhibits distinctive characteristics. It is closely linked to societal narratives surrounding national image, technological innovation and planetary sustainability [4,7], and is increasingly shaped by the privatization of space activities and the evolution of international space governance [8]. In spite of its strategic importance, space science communication discourse has not received enough attention so far. In view of the leading role of the U.S. and China in space exploration, this study gives a corpus-assisted discourse study of their space science communication on social media to examine their particular ways of positioning on social media. It has two objectives: (1) to compare their particular ways of positioning on social media; (2) to explain the socio-political factors behind their particular ways of positioning.

## 2. Space exploration in the U.S. and China

Both the U.S. and China are global leaders in space exploration, but they take different routes due to their historical contexts, cultural values and geopolitical priorities. The U.S. has a long history of space exploration, dating back to the Cold War space race with the Soviet Union [4]. NASA, founded in 1958, became the centerpiece of U.S. space activities, achieving milestones such as the Apollo moon landings, the Space Shuttle program, and the Mars rover missions [9]. The U.S. has consistently been a leader in space exploration, prioritizing scientific innovation, international collaboration, and private-sector involvement [10]. In contrast, China’s space program is relatively newer but has grown rapidly since its formal establishment in the 1950s under the China National Space Administration (CNSA) [11]. Amid growing strategic competition with the United States and its desire for national prestige [7], China’s space program has focused on achieving technological independence and enhancing its international influence in space activities [12]. Milestones such as the Chang’e lunar missions, the Tiangong space station, and the Tianwen-1 Mars mission showcase China’s rapid ascent in space exploration [13].

The U.S. has pursued space exploration with a mix of scientific, commercial, and diplomatic objectives. NASA’s Artemis program aims to return humans to the Moon and establish a sustainable presence as a stepping stone for future Mars missions [9]. The U.S. has also prioritized partnerships with private companies like SpaceX and Blue Origin, fostering innovation and reducing costs [3,14]. Additionally, the U.S. promotes international collaboration through programs like the International Space Station (ISS) and initiatives such as the Artemis Accords, which aim to set norms for peaceful exploration [15]. China’s space exploration strategy is closely tied to national pride, technological self-reliance, and geopolitical influence [7]. Its goals include building an independent human spaceflight program, conducting lunar and Mars exploration, and developing advanced satellite and space-based infrastructure [11]. China’s Tiangong space station, completed in 2022, symbolizes its ambition to rival the ISS and establish itself as a space power. While China has collaborated with Russia and other nations, its space activities are largely state-driven and independent of Western space alliances [11].

Therefore, the space programs of China and the United States reflect their respective priorities, values, and global ambitions. The U.S. leads in scientific innovation, international collaboration, and private-sector involvement [10,15], while China emphasizes state-driven development, national pride, and technological independence [12,13]. While competition between the two nations drives advancements in space exploration, their differing approaches and objectives highlight the diverse pathways toward humanity’s future in space.

## 3. Science communication and positioning

Science communication has moved away from one-way information delivery towards greater audience participation, with a greater emphasis on such outcomes as democratization, legitimation, innovation, education and inspiration [5]. Therefore, science communication has evolved from the deficit model, to the dialogic approach, and then the current participatory approach [16]. Recent studies have drawn attention to the mediatization of science and the participatory feature of science communication on social media. These studies have revealed how the formats, conventions and rules of social media shape the production and presentation of scientific content amplification [17].

However, disputes remain over how the participatory feature of science communication is realized on social media. Some studies argue that a significant number of official and institutional social media accounts for science communication still resort to a one-way communication format and often lack engagement, whereas some others even claim that information dissemination may, in certain contexts, achieve higher levels of interaction [18]. In addition, science communication is not a uniform practice but one closely tied to its specific contexts. Previous research shows that science communication is influenced by cultural variations grounded in different values and epistemological frameworks, exemplified by contrasts between European American and Native American communities [19] and by how societies frame responsibilities and risks related to climate change [20]. Furthermore, although social media has become the most widely used source of scientific information in most countries [21], research that approaches science communication from cultural or partly cultural perspectives has largely relied on news media as its primary data source. Studies examining such perspectives within social media contexts remain limited.

Previous studies have shown that several roles compete to shape science communication by government science agencies such as scientific institutions, public communicators, and government bodies. Their role as scientific institutions requires them to value credibility and authority, which can be established through scientific rigor and transparency [22]. Their role as public communicators enables them to bridge the gap between complex scientific information and the public, fostering engagement and understanding [23]. Their role as government bodies also requires them to align their scientific missions with national priorities, and construct discourses aimed at fostering public appreciation of science, justifying state investments in science and technology, and legitimizing government decisions [24]. While these roles can work synergistically, they also post significant challenges when they come into conflict. As a result, the particular ways of science communication can be viewed as a result of the contestation and negotiations between the expectations arising from these intertwined roles [25].

The concept of “positioning” is often used to understand the discursive construction of selves as an alternative to the more static or fixed idea of “role” in social psychology [26]. Adopting a particular position shapes how individuals perceive and interpret the world, as their understanding is influenced by the language, frameworks, and ideas relevant to the discourse tied to that position [26]. Building on this concept, positioning theory examines how language and discourse define the relative positions of individuals within social interactions. This involves the assignment, negotiation, and contestation of rights and obligations, as well as the assertion of claims for oneself and the imposition of duties on others [25]. Central to this theory is the recognition that participants in social interactions do not have equal access to the rights and duties required to perform certain meaningful actions. The distribution of these rights and duties shapes, and is reflected in, who can utilize specific “discourse modes” [27].

Positioning theory offers a robust theoretical lens for analyzing how government science agencies construct their “selves” in the context of space science communication. It acknowledges the profound influence of social contexts on how roles and norms are constructed. Rooted in insights on the social origins of higher mental processes, it highlights that mental functions first develop in social contexts before becoming internalized by individuals [28]. Besides, positioning theory emphasizes the agency of subjects in navigating and adopting roles within specific contexts [27]. It offers a normative framework for understanding how systems of meaning and social rules shape human actions, while also recognizing that individuals and institutions are not passive recipients of external forces. Instead, they actively choose which norms and roles to adopt based on the situational demands [27]. This dynamic perspective makes positioning theory especially valuable for analyzing how government science agencies manage competing expectations, balancing roles as educators, advocates, and representatives of state authority.

## 4. Corpus-assisted discourse studies

Critical discourse analysis (CDA) is a multidisciplinary approach which examines language use in its socio-political contexts and explores the relations between language, power, and ideology [29]. In science communication, CDA has proved especially effective in challenging the deficit model, which assumes a passive, uninformed public [30]. For example, previous CDA studies have demonstrated that passive constructions and nominalizations can conceal scientific agency and promote an image of science as value-neutral and objective [31]. Metaphors and pronoun choices have also been analyzed as key resources for managing epistemic authority and audience inclusion [32]. CDA has also shown how institutional actors employ conversationalization and synthetic personalization, such as direct address and an informal tone, to simulate proximity while maintaining asymmetrical control over interaction [33]. Although these strategies may enhance engagement, they risk undermining perceptions of scientific credibility or concealing top-down instrumental intentions [34].

Nonetheless, although CDA’s reflexive orientation requires researchers to examine their own positionality within the systems they study, it has been criticized for potential researcher bias and the selective use of examples [35]. In response, scholars increasingly integrate corpus linguistics (CL), a data-driven approach that employs computational tools to identify recurring patterns in large datasets [36]. This integration, known as corpus-assisted discourse studies (CADS), preserves the reflexive and context-sensitive strengths of CDA while enhancing empirical rigor and reproducibility through CL [37]. It helps mitigate confirmation bias by enabling systematic analysis of frequencies, collocations, and concordances across extensive corpora [35].

Corpus-assisted approaches offer effective tools for addressing key challenges in science communication: balancing rigor with accessibility, navigating ideological influences, and understanding audience responses. Corpus analyses reveal how linguistic features like hedges and boosters enhance clarity without sacrificing credibility [38] and how metaphors adapt to platforms and contexts to improve engagement [39]. Studies of public discourse, such as collocation patterns for terms like “vaccine”, highlight shared and culturally specific concerns [40], while analyses of user-generated content uncover how audiences co-construct scientific narratives [41]. Comparative corpus studies further show how national ideologies and cultural norms shape science discourse, with variations in thematic focus, metaphorical framing, and representations of expertise [32]. These insights emphasize the need for context-sensitive models of science communication.

## 5. Methodology

### 5.1 Data collection and corpus building

This study analyzes two corpora in Chinese and English, comprising social media posts on space exploration from government science agencies in China and the United States. Chinese-language content was collected from Weibo, a major platform in China known for its rich multimedia features, large user base (55% under 30), and role in government communication [42]. English-language content was collected from X (formerly Twitter), a global platform widely used by public institutions for updates, audience interaction, and real-time communication [43]. While Weibo and X differ in their regulatory environments, platform affordances, and communicative conventions, they provide a suitable basis for comparison because both are widely used by government institutions for public communication, and their functional comparability has been noted in previous research [44,45]. This study compares positioning strategies as they emerge within these comparable yet distinct platform contexts, while recognizing that platform-specific factors may influence certain discourse features.

From each platform, three verified accounts with the highest follower counts were selected for analysis, provided they met two specific criteria. First, the accounts needed to demonstrate clear relevance to national space exploration, ensuring that the content was directly aligned with the topic of interest. Second, the accounts were required to include official domain indicators, such as “gov.” or equivalent terms, in their descriptions, signifying formal institutional affiliation and credibility. These criteria ensured that the selected accounts represented authoritative sources of state-led science communication.

Based on these requirements, the selected Chinese accounts on Weibo include @中国航天科技集团 (China Aerospace Science and Technology Corporation), the primary contractor for China’s space program; @载人航天小喇叭 (Manned Space Loudspeaker), a channel dedicated to China’s crewed spaceflight updates and educational outreach; and @中国航天科工 (China Aerospace Science and Industry Corporation), a state-owned enterprise involved in space technology innovation. The English accounts selected from X (formerly Twitter) are @NASA, the official account of the United States’ space agency; @NASA’s Kennedy Space Center, the hub for launch operations; and @NASA’s Johnson Space Center, the center for human spaceflight and mission control.

Posts from these accounts were collected over a six-month period, from 1 April to 1 October 2023, to create two distinct corpora. All data used in this study were publicly available on the respective social media platforms at the time of collection, and the dataset was manually compiled by the researchers without accessing any private or restricted information, in compliance with the platforms’ terms and conditions. The Chinese-language corpus, sourced from Weibo, contains 895 posts comprising 24,769 tokens (equivalent to 67,926 characters in Chinese). The English-language corpus, compiled from X, includes 1,204 posts totaling 25,063 tokens (37,785 characters). This dataset offers a robust foundation for cross-linguistic and cross-cultural analysis of state-driven science communication regarding space exploration.

### 5.2 Data pre-processing and the text mining tool

The text mining tool KH Coder was used to process the data. Developed for quantitative content analysis and text mining [46,47], KH Coder integrates tools such as the Stanford POS Tagger, R, and MySQL enabling both linguistic and statistical processing [46]. The software supports multiple languages, including Chinese and English, and applies standardized co-occurrence measures such as the Jaccard index [47]. It provides researchers with advanced methods for analyzing large volumes of text data, including co-occurrence network analysis, correspondence analysis, hierarchical cluster analysis, and topic modelling [39]. As an open-source platform widely used in peer-reviewed research [e.g., 48,49,50], KH Coder enhances methodological transparency and reproducibility in large-scale textual analysis.

Before the formal corpus analysis, the data underwent preprocessing. English texts were lemmatized, and both the English and Chinese corpora were automatically segmented and then manually checked. In KH Coder, lexical items are stored at the level of base forms (lemmas) while surface forms are retained in the database, which helps keep subsequent frequency and co-occurrence analyses traceable and reproducible [51]. Segmentation accuracy was checked in three stages. First, both corpora were imported into KH Coder and raw frequency lists were generated. Second, these lists were reviewed to identify potential segmentation errors. Two types of errors were most common. In the Chinese-language corpus, some social media expressions and aerospace-specific terms not included in standard dictionaries were incorrectly split, such as “航小科” (*Hangxiaoke*, a mascot-based self-addressing) and “晨语” (*morning greetings*). In the English-language corpus, some multi-word expressions functioning as single units, such as project names like “Artemis II”, were also split. Third, these errors were corrected using the “Select Words to Analyze” function, with relevant items manually entered as complete tokens. After these adjustments, a revised token frequency list was generated to confirm that correctly segmented tokens were retained.

### 5.3 Analytical framework and procedure

This study draws on positioning theory, which links positioning to storyline, illocutionary force, and rights and duties [52], as well as critical discourse analysis, particularly the ideational, relational, and identity functions of discourse [53]. Although positioning can be realized at different levels of discourse, this study proposes to examine the particular ways of positioning on social media in terms of three language features in discourse: (1) topics/themes, (2) addressing terms, and (3) co-occurrence patterns of self-addressing terms. These language features provide observable indicators through which discursive positioning can be examined in the corpus. By selecting and emphasizing specific themes, discourse signals values, priorities, and expertise, shaping what is considered important and influencing audience perceptions of the communicator. Addressing terms, such as those for individuals, organizations, or events, further contribute to positioning by constructing roles, responsibilities, and relationships. These choices enable communicators to assert authority, build closeness with audiences, or create distance. Similarly, self-addressing terms and these words that co-occur with self-addressing terms can shape identity and audience alignment, influencing perceptions of authority, inclusivity, and responsibility. With corpus assistance, analyzing these linguistic resources allows the study to trace how government science agencies establish their position within the discourse of space science communication. This analytical approach follows Fairclough [53]’s socio-dialectical perspective, which treats discourse as operating simultaneously at the levels of text, discursive practice, and social practice.

With KH Coder, this study begins with a co-occurrence network analysis. Based on statistical measures such as the Jaccard coefficient [46], the software identifies words that have a strong tendency to co-occur with each other within defined textual units (e.g., sentences or paragraphs), and visualizes these patterns as networks in which nodes represent terms and edges (connections) indicate their co-occurrence. In the present study, co-occurrence was calculated with post-level text segments (H5 units in KH Coder) as the unit of analysis. Tokens were filtered using a minimum term frequency threshold of 30, and the network visualization was generated from the top 150 co-occurrence edges ranked by the Jaccard coefficient. KH Coder groups related terms into color-coded clusters; solid lines indicate links between words within the same cluster, whereas dashed lines indicate links between different clusters [51]. They can suggest the topics/themes that are prominent in a corpus. The different ways of positioning can be perceived through their preferences for different topics/themes.

It is followed by a detailed examination of three groups of addressing terms: self, the public, and the nation. The process begins with identifying the terms that fall into each of these three groups, as well as exploring the different ways in which these groups are linguistically realized in the discourse [e.g., 54]. All posts in the corpus were reviewed to determine the specific words, phrases, and pronouns used to refer to these groups. By examining these variations, the study reveals how these groups are conceptualized and addressed in different communicative contexts. Following this identification, the frequencies of these addressing terms are systematically counted and compared across the corpus. This quantitative analysis highlights the relative prominence of each group and sheds light on which addressing terms are prioritized or emphasized. By analyzing these addressing terms, the study uncovers how different groups are categorized, labelled, and framed within the discourse. The choice of these addressing terms is key to understanding their preferential ways of positioning themselves and others.

Finally, self-addressing terms were examined using KH Coder’s word-association function. In each corpus, self-addressing terms were coded as a distinct category following KH Coder’s coding rules. A word association analysis was performed to examine those words that have a strong tendency to co-occur with each other in a sentence. The strength of co-occurrence was measured by the Jaccard coefficient, and the network visualization displays the top 150 strongest co-occurrence relationships. In the network, boxed nodes mark the query terms (shown as double rectangles in KH Coder), while related terms are grouped into color-coded clusters; solid lines indicate links within a cluster and dashed lines indicate links between clusters [51]. By examining these co-occurring words in context, the analysis considers how self-addressing operates as a coherent conceptual grouping rather than as isolated lexical items (Higuchi, 2017b). these strong co-occurring words of self-addressing terms can reveal the specific strategies that are used to position themselves.

A tripartite analysis of the three types of linguistic resources at different levels of discourse allows us to triangulate our findings regarding their preferential ways of positioning. These findings are further interpreted through the lens of social, geopolitical, and platform contexts to gain a comprehensive understanding of their positioning strategies. This study is “critical” in this sense that it tries to explain their preferential ways of positioning in relation to their specific contexts of use, while uncovering the underlying social, geopolitical, and platform-related factors that shape them [55]. Importantly, this study does not assume that one approach to positioning is inherently better than another. Instead, we argue that particular ways of positioning are valid and effective as long as they fulfill their intended communication purposes in their respective contexts.

Nevertheless, the contextual interpretation requires reflexivity regarding the researchers’ own positionality. As complete neutrality is neither attainable nor expected in cross-cultural discourse analysis, reflexive acknowledgment of positionality is widely recognized as a way to strengthen analytical credibility [31,55]. It is important to acknowledge that the researchers’ identities as native Chinese speakers might influence the understanding and interpretation of self-positioning on X (formerly Twitter) and Weibo in this study. To minimize this impact, we aim to make the analytic procedures and results as explicit and replicable as possible, subjecting them to critical assessment by readers from diverse socio-political backgrounds.

## 6. Findings

### 6.1 Representations of space exploration

**Fig 1:**
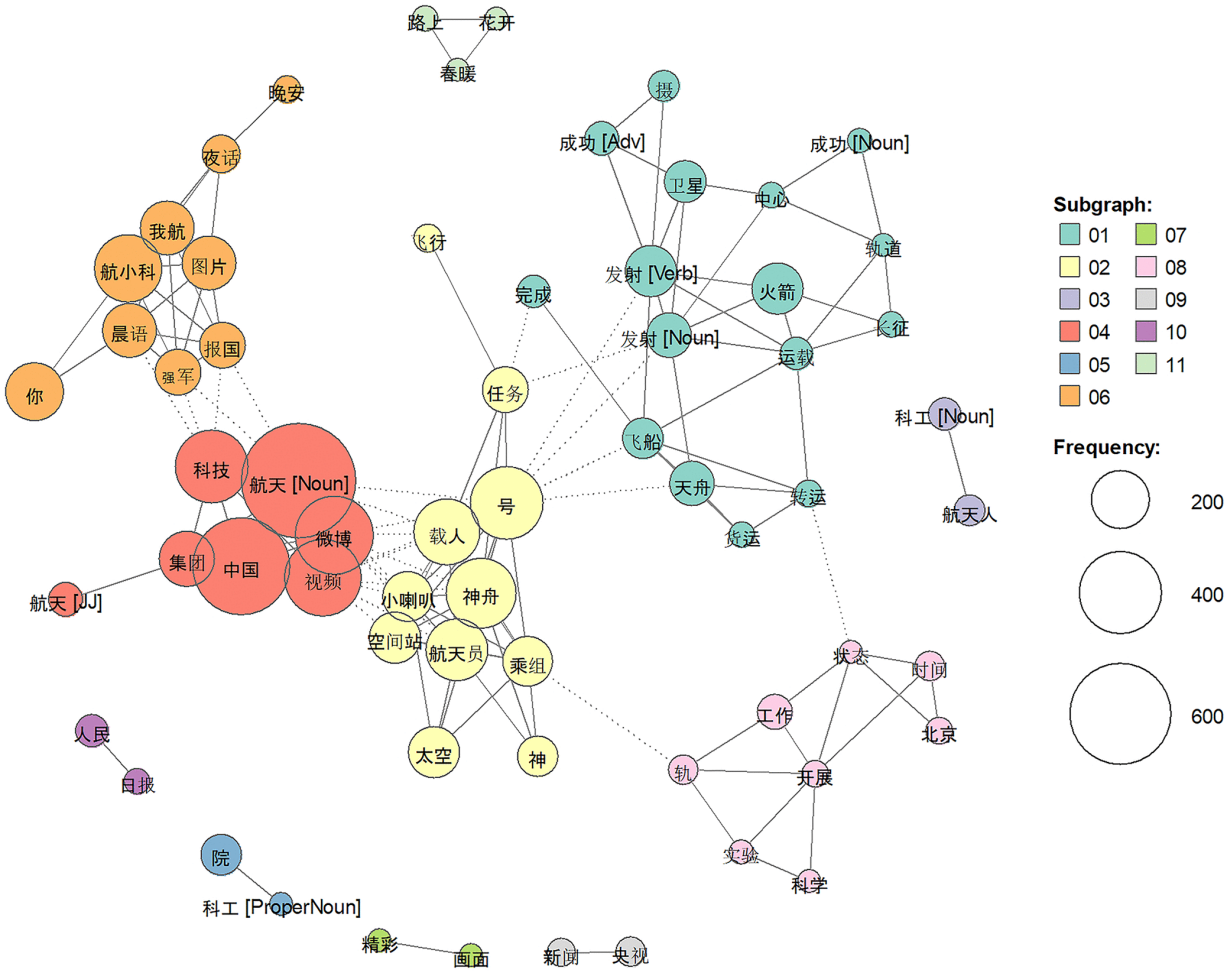
Co-occurrence network showing five prominent themes in the Chinese-language corpus (see S1 Table for English translations of the tokens).

**Fig 2:**
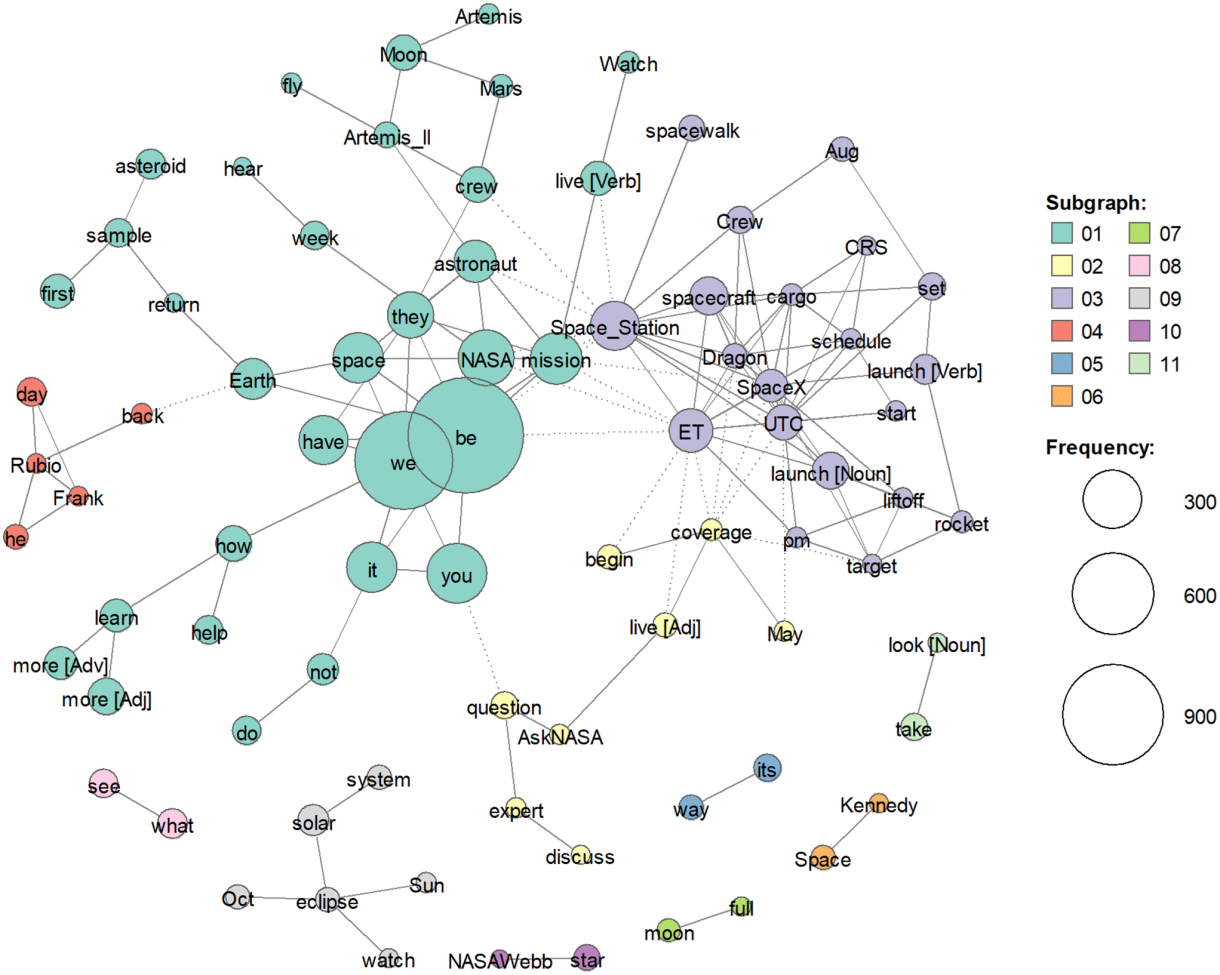
Co-occurrence network showing five prominent themes in the English-language corpus.

Figures 1 and 2 present the co-occurrence networks of the two corpora, with five prominent themes emerging in the Chinese-language corpus. **Theme 1** (**Cluster 1**) celebrates specific milestones, particularly rocket launches. Keywords such as “长征” (*Long March*), “火箭” (*rocket*), “卫星” (*satellite*), “任务” (*mission*), and “成功” (*successfully*) highlight technical achievements and mission outcomes, pointing to an emphasis on operational capability in space exploration. **Theme 2** (**Cluster 2**) emphasizes the human and infrastructural elements of China’s space program. Terms such as “神舟” (*Shenzhou*), “空间站” (*space station*), “乘组” (*crew*), and “航天员” (*astronauts*) spotlight the people and technologies that enable space missions. By showcasing astronauts and high-profile projects, this cluster highlights the human effort and technological sophistication underlying China’s achievements. **Theme 3** (**Cluster 4**) reflects the institutional nature of China’s space science communication. Tokens such as “中国” (*China*), “航天” (*aerospace*), “科技” (*science and technology*), and “集团” (*Group*) signal formal organizational affiliations, while references to “微博” (*Weibo*) and “视频” (*video*) indicate the use of official social media channels for communication. **Theme 4** (**Cluster 6**) blends personalization with patriotic sentiment, creating a relatable and engaging voice for institutional accounts. Phrases like “我航” (*our team*), “航小科” (a self-addressing term used by China Aerospace Science and Industry Corporation), “晨语” (*morning words*), and “晚安” (*good night*) convey warmth and approachability. These messages are often paired with patriotic expressions like “报国” (*serving the nation*) and “强军” (*strengthening the army*), reinforcing a sense of national pride and collective identity. This cluster personalizes institutional messaging, fostering emotional connection with the audience. **Theme 5** (**Cluster 8**) focuses on the operational and logistical aspects of space missions, with tokens such as “北京” (*Beijing*), “时间” (*time*), “状态” (*status*), “开展” (*carry out*), and “实验” (*experiment*). This cluster highlights the systematic execution of tasks, reflecting the precision and professionalism of China’s space program. By emphasizing technical details, this theme positions space exploration as a rigorously planned and executed endeavor.

The English-language corpus presents five themes that likewise deserve attention. **Theme 1 (Cluster 1)** focuses on institutional and participatory mission narratives. Tokens combine the neutral institutional label *NASA* with personal pronouns such as *we*, *you,* and *they*, alongside mission references including *Artemis_II*, *Artemis*, and celestial bodies such as *Moon*, *Mars* and *asteroid*. Interactive expressions like *learn*, *watch, live,* and *hear* further invite audience participation. Tokens of this cluster represent space missions as shared endeavors, positioning the agency and the audience within a common narrative of exploration. **Theme 2 (Cluster 2)** highlights audience engagement. Terms such as *AskNASA*, *question*, *discuss*, and *expert* point to efforts to encourage interaction and dialogue with audiences, alongside references to *live* and *coverage*, which indicate both real-time and pre-produced forms of communication. These patterns suggest that U.S. agencies seek to make scientific information more accessible and to involve audiences in the discussion of space science. **Theme 3 (Cluster 3)** centers on mission execution and coordination. Tokens such as *rocket*, *satellite*, *liftoff,* and *spacewalk*, together with temporal markers including *UTC*, *pm*, and *Aug*, situate space activities within scheduled operational timelines. At the same time, references to *Crew*, *Space_Station*, *Dragon*, and *CRS*, along with commercial actors such as *SpaceX*, highlight the coordination of personnel, infrastructure, and private partners. These tokens depict space missions as time-regulated and collaboratively managed operations. **Theme 4 (Cluster 4)** highlights individual astronauts, with tokens such as *Rubio* and *Frank*, and references to return-related terms such as *back*. **Theme 5 (Cluster 9)** centers on specific celestial bodies and astronomical events. Tokens such as *eclipse*, *solar*, *system*, and *Sun*, together with time markers like *Oct*, point to references to observable space events. These elements indicate efforts to inform the public about ongoing celestial phenomena while also inviting interest and curiosity.

Overall, the Chinese-language corpus presents a cohesive discourse that intertwines technical achievements, personnel and infrastructural capacity, and operational execution with expressions of patriotic sentiment and collective pride, while signaling its state-affiliated character and incorporating moments of emotional warmth. In contrast, the English-language corpus combines mission execution, operational infrastructure, coordination with commercial partners, and audience engagement within institutional and participatory narratives that present space exploration as a shared endeavor. Compared with the collective and state-centered emphasis in the Chinese-language corpus, individual astronauts are more visibly referenced in the English-language corpus, alongside specific celestial bodies and observable astronomical events.

### 6.2 Overall analysis of addressing terms

**Fig 3:**
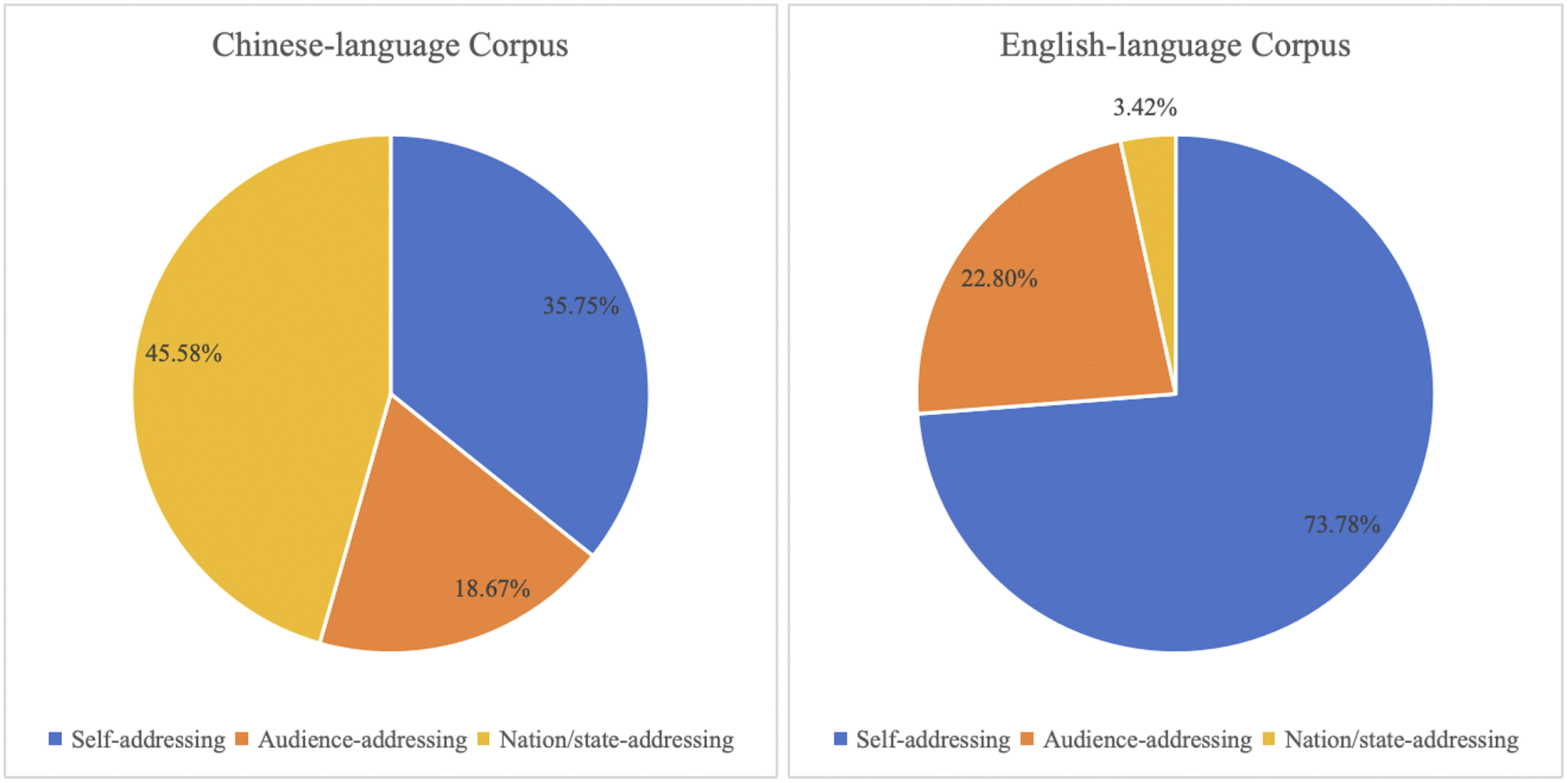
Distribution of addressing terms in the two corpora.

Figure 3 presents the addressing terms identified in the Chinese and U.S. corpora, grouped into three categories: self-addressing (35.75% in the Chinese-language corpus; 73.78% in the English-language corpus), audience-addressing (18.67% in the Chinese-language corpus; 22.80% in the English-language corpus), and nation/state-addressing terms (45.58% in the Chinese-language corpus; 3.42% in the English-language corpus) (see Tables 1 and 2).

**Table 1:** Addressing terms used in the Chinese-language corpus.

Types	Terms	Counts	%
Self-addressing	航小科(*Hangxiaoke*, a mascot-based form of self-addressing)	272	18.07%
	我航 (*Wohang*)	171	11.36%
	我们 (*we*)	86	5.71%
	国家队(*National Team*)	7	0.47%
	咱们 (colloquial *we*)	2	0.13%
	Subtotal	538	35.75%
Audience-addressing	你 (*you*)	204	13.55%
	大家 (*everyone*)	34	2.26%
	你们 (*you all)*	10	0.66%
	考生 (*test-takers*)	8	0.53%
	网友(*netizens)*	7	0.47%
	小朋友(*little ones*)	7	0.47%
	同学们(*classmates*)	4	0.27%
	小伙伴们(*dear friends*, informal, youthful tone)	2	0.13%
	各位(*everyone*)	1	0.07%
	吃货(*foodies*, informal)	1	0.07%
	朋友们 (*friends*)	1	0.07%
	家人们(*family members*)	1	0.07%
	童鞋们(*tongxiemen*,a misspelling of “students”)	1	0.07%
	Subtotal	281	18.67%
Nation/state-addressing	中国 (*China*)	580	38.54%
	我国(o*ur country*)	64	4.25%
	祖国(*motherland*)	40	2.66%
	中华民族(*Chinese nation*)	2	0.13%
	Subtotal	686	45.58%
Total		1505	100%

**Table 2:** Addressing terms used in the English-language corpus.

Types	Terms	Counts	%
Self-addressing	*we*	389	38.06%
	*NASA*	247	24.17%
	*us*	118	11.55%
	Subtotal	754	73.78%
Audience-addressing	*you*	218	21.33%
	*everyone*	4	0.39%
	*skywatchers*	3	0.29%
	*digital creators*	2	0.20%
	*content creators*	2	0.20%
	*members of the media*	2	0.20%
	*metalheads*	1	0.10%
	*listeners*	1	0.10%
	Subtotal	233	22.80%
Nation/state-addressing	*U.S.*	26	2.54%
	*US*	7	0.68%
	*United States*	2	0.20%
	Subtotal	35	3.42%
Total		1022	100%

Self-addressing terms reveal how the social media accounts of each country’s government science agencies refer to themselves. In the Chinese-language corpus, expressions such as “航小科” (*Hangxiaoke*) suggest efforts to present the agency in a more approachable manner. Terms like “我航” (*Wohang*) and “咱们” (colloquial *we*) convey solidarity through inclusive language, fostering a sense of connection with followers. The expression “国家队” (*National Team*) positions the agency as part of a collective national effort and is also used metaphorically to signal state-level prestige and official representation. Self-addressing terms employed in the Chinese-language corpus blend state-affiliated character with interpersonal closeness and collective identity, as demonstrated in Excerpt 1. By contrast, the English-language corpus relies primarily on *we*, *NASA*, and *us.* Although self-addressing terms account for a higher proportion of the English-language corpus than in the Chinese-language corpus, they involve a more limited range of lexical variants. These accounts refer to themselves through standard pronouns and the formal label *NASA*, adopting a relatively neutral mode of self-addressing. Unlike the varied and colloquial expressions observed in the Chinese-language corpus, the consistent use of *NASA* contributes to a stable and recognizable institutional identity, as demonstrated in Excerpt 2.

Excerpt 1:

#航小科晨语# 将你梦藏在心里，行动落于脚下，用柔软的眼光看世界，用温暖的心面对生活。早安 #科技强军 航天报国# （图片来自我航于小鱼）

(May 27, 2023)

English translation: #MorningWordsFromHangxiaoke# Keep your dreams in your heart and turn them into action. See the world with gentle eyes and face life with a warm heart. Good morning. #StrengthenTheMilitaryWith ScienceAndTechnology ServeTheNationThroughAerospace# (Photo credit: Xiaoyu from our aerospace team)

Excerpt 2:

This week at NASA: The @NASAArtemis II astronauts visit the spacecraft that will take them around the Moon, teams practice recovery for the #Artemis II splashdown, and @NASAWebb takes another look at the most distant star ever detected. Subscribe: http://nasa.gov/subscribe

(August 12, 2023)

Audience-addressing terms reveal how the accounts engage their followers. The Chinese-language corpus contains 13 distinct terms, compared to 8 in the English-language corpus, indicating a wider range of audience categories (as shown in Tables 1 and 2). In both corpora, the second-person pronoun “你” (*you*) or “*you*” is the most frequently used term. However, the Chinese-language corpus also employs more specific expressions such as “考生” (*test-takers*), “网友” (*netizens*), “小朋友” (*little ones*), “同学们” (*classmates*), and “朋友们” (*friends*). These terms construct diverse audience identities and establish intimacy with different segments of followers, including students and general audiences. They also suggest an educational orientation, reflecting a perceived responsibility to communicate scientific knowledge to children and adolescents. Informal expressions such as “小伙伴们” (*dear friends*) and “童鞋们” (*tongxiemen*, a playful misspelling of *students*) further personalize the tone, positioning the account both as a mentor and as an approachable peer. By contrast, audience-addressing terms in the English-language corpus follow a more neutral and professional pattern of address. Expressions such as *you*, *everyone*, *listeners*, and *members of the media* avoid endearing form. At the same time, targeted terms such as *skywatchers*, *digital creators*, and *content creators*, imply an audience with an existing interest in space and a certain level of familiarity with scientific topics. This pattern suggests a more interest-based segmentation of the public, oriented toward expertise and media participation rather than demographic attributes such as age.

Nation/state-addressing terms showcase how each corpus aligns with national identity. The Chinese-language corpus places strong emphasis on this category, with terms such as “中国” (*China*), “我国” (*our country*), “祖国” (*motherland*), and “中华民族” (*Chinese nation*) accounting for 45.58% of all addressing terms. The frequent use of expressions such as “祖国” and “我国” reflects a strong alignment with national pride and collective identity. This pattern links space achievements to national accomplishment and underscores collective belonging, presenting space exploration as a common achievement of the nation, as demonstrated in Excerpt 3. By contrast, nation/state-addressing terms are relatively limited in the English-language corpus, accounting for 3.42% of the total. The most frequently used forms are *U.S.*, *US*, and *United States*, which occur far less often than their Chinese counterparts. The corpus does not include affective expressions comparable to “祖国” (*motherland*). References to the nation remain largely formal, while expressions of pride and evaluation are more often associated with mission history, crew achievement, and future exploration rather than collective national identity, as illustrated in Excerpt 4.

Excerpt 3:

【从青藏高原到渤海，空间站跨越祖国】神舟十六号航天员乘组从中国空间站舷窗拍摄到祖国三级阶梯的极致之美。从高耸巍峨的高原山脉，到茫茫苍黄的黄土地，再到原野寥廓的华北平原，从航天员的视角看到的祖国大地震慑人心。#第一视角从中国空间站看地球# #天宫TV第五季# 载人航天小喇叭的微博视频

(September 26, 2023)

English translation: [From the Tibetan Plateau to the Bohai Sea — the space station crosses our homeland.] The Shenzhou-16 crew captured breathtaking images of China’s three-step terrain from the window of the China Space Station. From towering plateau mountains, to the vast loess lands, and then to the expansive North China Plain, the view of our homeland from space is truly awe-inspiring. #EarthFromTheSpaceStation #TiangongTVSeason5 Video via Manned Spaceflight Updates (Weibo)

Excerpt 2:

50 years ago today, the first crew launched to the first U.S. space station: Skylab. Skylab proved that humans could live & work in low gravity while staying healthy. Its legacy spurs us to innovate ways that keep crew safe & mission-ready on trips to the Moon, Mars, & beyond!

(May 25, 2023)

In sum, addressing terms reveal how government science agencies position themselves in relation to the audience and the nation, and the two corpora display distinct patterns in this respect. The Chinese-language corpus employs a wider range of addressing terms across all three categories, combining explicit national references with inclusive and approachable forms of address. This links state affiliation with collective identity while maintaining interpersonal closeness. By contrast, the English-language corpus relies on a more limited and standardized set of self- and audience-addressing forms. Audience-addressing tends to be neutral or interest-based, while nation/state-addressing remain formal. Expressions of pride tend to center on mission achievement and exploration rather than collective national belonging.

### 6.3 Analysis of self-addressing terms

**Fig 4:**
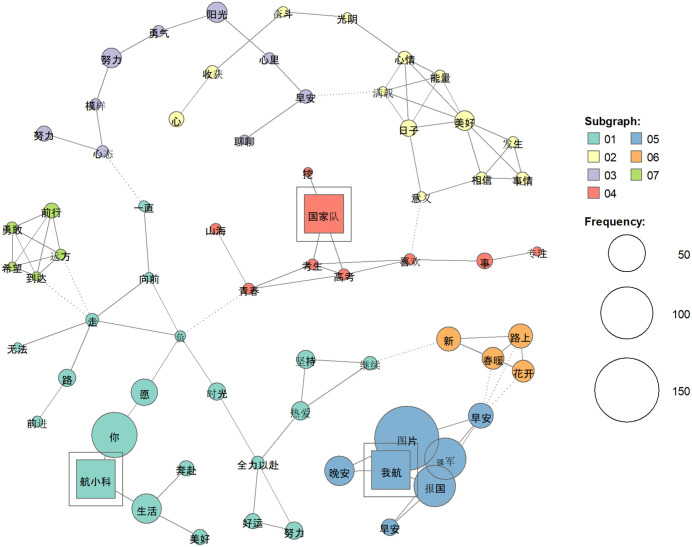
Co-occurrence network of self-addressing terms in the Chinese-language corpus (see S2 Table for English translations of the tokens).

**Fig 5:**
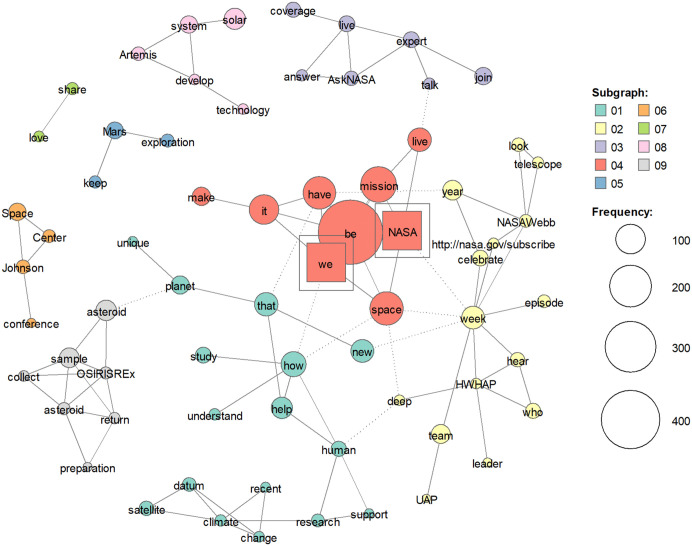
Co-occurrence network of self-addressing terms in the English-language corpus.

Figures 4 and 5 show clusters associated with self-addressing terms in both corpora and reveal differences in how government science agencies articulate their rights and responsibilities in the two national contexts.

In the Chinese-language corpus, self-addressing terms co-occur with words conveying motivation, encouragement, and emotional support for followers. For instance, **Cluster 1** includes terms like “航小科” (*Hangxiaoke*), “你” (*you*), “生活” (*life*), “全力以赴” (*spare no effort*), “愿” (*wish*), and “美好” (*wonderful*), which convey hopes for followers to work hard and achieve a brighter future. **Cluster 2** features words such as “美好” (*wonderful*), “日子” (*days*), “奋斗” (*strive*), and “收获” (*gain*), emphasizing living a meaningful and fulfilling life. **Cluster 3** focuses on morning greetings and motivational messages, with terms like “早安” (*good morning*), “勇气” (*courage*), and “心态” (*mindset*), encouraging followers to work hard for their goals. **Cluster 4** is tailored to students, with terms like “国家队” (*national team*), “考生” (*examinees*), and “高考” (*National Entrance Exam*), expressing wishes for success in exams and the pursuit of one’s passions. **Cluster 5** includes greetings such as “晚安” (*good night*) and “早安” (*good morning*) as well as nationalistic terms like “报国” (*serve the nation*) and “强军” (*strengthen the military*), suggesting an effort to build closeness with followers through both everyday greetings and appeals to patriotic sentiment. **Cluster 6** uses metaphorical language like “春暖” (*warm spring*) and “花开” (*flowers bloom*) to symbolize a beautiful life journey and inspire positivity. **Cluster 7** includes terms such as “前行” (*move forward*), “勇敢” (*brave*), and “希望” (*hope*), highlighting the importance of courage and perseverance in achieving distant goals. These clusters collectively present the agency as a supportive and motivational figure, while encouraging followers to see themselves as hardworking individuals striving for self-improvement and collective cohesion.

In contrast, in the English-language corpus, self-addressing terms co-occur with tokens conveying references to celestial bodies and scientific phenomena, names of space exploration programs and infrastructure, and invitations to audience engagement. **Cluster 1** includes words like *planet*, *study*, *climate*, and *research*, indicating attention to planetary research and climate-related topics. **Cluster 2** features terms like *telescope*, *NASAWebb*, and *learn*, reflecting communication around scientific discovery and observational projects. **Cluster 3** emphasizes engagement, with words such as *coverage*, *live*, *AskNASA*, and *expert*, inviting followers to participate in live events and interact with experts. **Cluster 4** focuses on space missions with terms like *mission*, *space*, and *live*, while **Cluster 5** highlights Mars exploration with words like *Mars* and *exploration*. **Cluster 6** includes references to *Space*, *Center*, *Johnson*, and *conference*, pointing to institutional events and activities. **Cluster 7** contains words like *love* and *share*, suggesting expressions of enthusiasm for sharing discoveries. **Clusters 8** and **9** focus on technological advancements and extraterrestrial exploration, with terms such as *solar*, *technology*, *Artemis*, *asteroid*, *sample*, and *return*. These clusters collectively present the agency as a source of exploration updates and scientific information, while encouraging followers to participate actively in space science communication.

In summary, the co-occurrence patterns of self-addressing terms in the Chinese-language corpus presents the agency as an experienced and supportive guide, offering value guidance and inspiration, while also extending encouragement, greetings and well wishes. By contrast, the co-occurrence patterns in the English-language corpus presents the agency as a central scientific actor on the global stage of space exploration, leading space programs and serving as an authoritative source of new findings, while also providing updates on space science to the public as part of its responsibility.

## 7. Discussion and conclusion

By constructing two corpora from the social media communication of government science agencies in China and the U.S., and by examining (1) thematic content, (2) forms of address, and (3) tokens co-occurring with self-addressing terms, this study explores how these agencies represent space exploration, define their relationship with the public and the nation, and articulate their institutional roles and responsibilities. Drawing on critical discourse analysis and positioning theory [27,53], these dimensions are examined as elements of discursive positioning within specific socio-cultural contexts, international environments and social media platform conditions.

As revealed above, Chinese government science agencies present themselves as state-affiliated scientific institutions that are also approachable, combining achievements and operational capacity with patriotic expression and collective pride. A wider range of addressing terms and self-addressing co-occurrence patterns construct the agency as an experienced and supportive guide, suggesting efforts to balance multiple expectations while linking national identity with interpersonal closeness. By contrast, U.S. government science agencies present themselves as professional scientific institutions focusing on research and space exploration, integrating mission execution, infrastructure, commercial coordination and audience engagement, with little emphasis on explicit state affiliation. Addressing terms are more standardized, with national references remaining formal and pride centering on mission performance and discovery. Co-occurrence patterns highlight expertise and program updates, emphasizing scientific leadership.

The differences observed are not merely linguistic choices but semiotic resources through which agencies construct self-positioning and negotiate legitimacy, authority and audience engagement within specific social, geopolitical and platform contexts. In social context where collectivism is culturally salient, and where power distance remains relatively high, proximity to state authority can reinforce institutional credibility [56]. The Chinese-language corpus reflects this pattern through frequent nation-addressing terms and the coupling of mission development with national milestones and collective belonging. Where space exploration is primarily state-funded [11], linking mission success to national pride may also function as a form of public legitimation tied to collective resource mobilization. At the same time, the wide range of addressing terms suggests an effort to accommodate diverse audience expectations and foster engagement in a social context that emphasizes solidarity and harmony [57]. Daily greetings and encouragement help draw audiences closer, and in some instances extend to expressions of value orientation and collective aspiration. Rather than constituting symmetrical dialogue, they may be understood, following the notion of “synthetic personalization”, as simulated intimacy within a communicative structure shaped by relatively high power distance [53]. The pattern thus maintains state-linked authority while softening its presentation in the social media context. In lower power-distance contexts, authority and expert claims are more likely to be challenged [58]. In such settings, overt alignment with state authority may not enhance credibility and can, in some cases, invite skepticism regarding scientific neutrality. Although NASA is formally a government agency, references to the nation in the corpus remain limited and largely formal. This relative deemphasis on national affiliation may shift attention toward professional expertise and scientific output as sources of legitimacy. Providing scientific evidence, including data, discoveries and program updates, can therefore be understood as a way of sustaining credibility in contexts where authority and expertise are subject to challenge and where science may be perceived as politically influenced [24]. In addition, where commercial partnerships and diversified funding structures are integral, signaling expertise and procedural detail may function as ways of demonstrating transparency valued in market-oriented environments [14]. The relatively neutral and interest-based addressing patterns, together with limited emotional national references, are consistent with this institutional positioning.

These differences can also be viewed in the wider global context, where space capability is often seen as a marker of national strength and prestige [4]. Although both China and the United States are leading spacefaring nations, their strategic priorities differ. As a more recent entrant that has progressed rapidly, China continues to emphasize independent capability and national development [11]. Linking space achievements to collective identity may strengthen domestic cohesion and highlight state-led progress, and the discourse appears largely inward-focused. The United States has a longer history in space exploration and operates within established international and commercial networks [59]. In this environment, government science agencies present themselves as relatively autonomous scientific institutions. Emphasizing mission outcomes, scientific findings and program updates, while limiting overt national references, may be better suited to cross-national collaborative settings and reduce the likelihood that communication is interpreted primarily through political or governmental lenses, particularly in environments sensitive to the politicization of science.

The influence of social media on government science agencies’ self-positioning appears context-specific, as social media affordances, while expanding reach and interaction, do not necessarily generate symmetrical dialogue or greater democratization of discourse [60]. On Weibo, official accounts operate within a regulated environment where content is subject to governance and review [61]. It is therefore unsurprising that space exploration missions are frequently presented as collective national achievements linked to patriotism and unity. Regulation, however, does not eliminate competition for attention. The wider range of addressing terms and affective expressions in the Chinese-language corpus may reflect agencies’ attempts to broaden appeal and engage diverse audience segments, indicating that even state-affiliated accounts must compete for visibility. In this sense, audiences constitute a form of influence that shapes science communication discourse. Social media does not fundamentally alter authority but reshapes how it is expressed, often in softer and more emotionally engaged forms. On X, communication takes place in a more openly contestable space where institutional claims can be publicly challenged [62]. Hashtags such as “AskNASA” are particularly noteworthy. Beyond signaling responsiveness to participatory norms characteristic of social media logic, they allow users to exercise a degree of “topic control” [53], shifting some initiative to the audience, although questions are selected and responses curated. At the same time, the consistent use of scientific terminology, mission updates and references to celestial bodies presupposes an audience capable of interpreting specialized information. The predominance of self-addressing terms and interest-based audience categories further indicates a focus on institutional expertise and audience segmentation. Participation is enabled, but it remains structured by knowledge expectations and institutional selection.

By incorporating text mining into critical discourse analysis, this study proposes a multi-level framework for examining how government science agencies position themselves on social media in relation to social context, the international environment and platform conditions. As space exploration attracts growing global attention, agencies may face increasing pressure to balance national narratives with international cooperation. Understanding institutional self-positioning therefore supports more critical awareness of the priorities embedded in scientific information. The tension between authority, legitimacy and participatory expectations is likely to remain a continuing focus to future research on space science communication, to which this framework aims to contribute.

Nevertheless, there are various linguistic resources at multiple levels of discourse contributing to positioning on social media language use. This study only addresses three representative linguistic resources, and future studies can examine more implicit linguistic resources that are key to positioning on social media language use. Furthermore, with the development of computer science and generative AI, future studies can draw on more advanced and efficient analytic tools and methods for the efficient and accurate identification and analysis of positioning on social media language use. It is expected that this study can lead to more studies towards this endeavor.

## Supporting information

S1 Table English translations of Chinese tokens in the clusters shown in Fig 1 (Chinese-language corpus co-occurrence network). (DOCX)

S2 Table English translations of Chinese tokens in the clusters shown in Fig 4 (self-addressing co-occurrence network in the Chinese-language corpus). (DOCX)

S1 Data Constructed corpora for this study. (XLSX)
