# Supplementary material for: Self-positioning in space science communication: A corpus-assisted discourse study
Source: PLoS One. 2026 Jul 9;21(7):e0353260. doi: 10.1371/journal.pone.0353260 (PMC13349144; doi:10.1371/journal.pone.0353260)
Supplement: S1 Table — (DOCX) [file pone.0353260.s001.docx]

S1 Table. English translations of Chinese tokens in the clusters shown in Fig 1 (Chinese-language corpus co-occurrence network).

| Clusters | Tokens |
| --- | --- |
| 1 | 发射(launch), 发射 (launch), 火箭 (rocket), 成功 (success), 成功 (successfully), 摄 (capture), 卫星 (satellite), 中心 (center), 轨道 (orbit), 长征 (Long March), 运载 (carry), 转运 (transfer), 货运 (cargo transport), 天舟 (Tianzhou), 飞船 (spacecraft), 完成 (complete) |
| 2 | 神舟 (Shenzhou), 空间站 (space station), 航天员 (astronaut), 小喇叭 (loudspeaker), 乘组 (crew), 太空 (outer space), 号 (mission designation), 载人 (manned), 任务 (mission), 飞行 (flight) |
| 3 | 科工 (aerospace industry), 航天人 (aerospace professionals) |
| 4 | 中国 (China), 航天 (aerospace), 航天 (aerospace), 微博 (Weibo), 视频 (video), 科技 (technology), 集团 (group) |
| 5 | 科工 (CASIC), 院 (academy) |
| 6 | 航小科 (Hangxiaoke, , a mascot-based form of self-addressing), 我航 (Wohang, our aerospace program), 图片 (image), 晨语 (morning message), 强军 (strengthen the military), 报国 (serve the nation), 你 (you), 夜话 (night talk), 晚安 (good night) |
| 7 | 精彩 (wonderful), 画面 (scene) |
| 8 | 开展 (carry out), 科学 (science), 实验 (experiment), 轨 (orbit), 工作 (work), 状态 (status), 北京 (Beijing), 时间 (time) |
| 9 | 新闻 (news), 央视 (CCTV) |
| 10 | 人民 (people), 日报 (daily newspaper) |
| 11 | 路上 (on the road), 花开 (flowers bloom), 春暖 (warm spring) |
