# Supplementary material for: Self-positioning in space science communication: A corpus-assisted discourse study
Source: PLoS One. 2026 Jul 9;21(7):e0353260. doi: 10.1371/journal.pone.0353260 (PMC13349144; doi:10.1371/journal.pone.0353260)
Supplement: S2 Table — (DOCX) [file pone.0353260.s002.docx]

S2 Table. English translations of Chinese tokens in the clusters shown in Fig 4 (self-addressing co-occurrence network in the Chinese-language corpus).

| Clusters | Tokens |
| --- | --- |
| 1 | 航小科 (Hangxiaoke), 你 (you), 生活 (life), 愿 (wish), 奔赴 (set out for), 美好(the good things), 全力以赴(go all out), 好运 (good luck), 努力 (make efforts), 热爱 (passion), 坚持 (persevere), 继续 (continue), 一直 (always), 向前 (move forward), 负 (bear), 时光 (time), 走 (walk), 无法 (cannot), 路 (road), 前进 (advance) |
| 2 | 美好 (beautiful), 日子 (days), 发生 (happen), 相信 (believe), 事情 (things), 意义 (meaning), 心情 (mood), 满载 (be filled with), 能量 (energy), 光阴 (time), 奋斗 (strive), 收获 (gain), 心 (heart) |
| 3 | 早安 (good morning), 聊聊 (chat), 心里 (in the heart), 阳光 (sunshine), 勇气 (courage), 努力 (effort), 模样 (appearance), 心态 (mindset), 努力 (make efforts) |
| 4 | 国家队 (national team), 山海 (mountains and seas), 青春 (youth), 考生 (examinees), 高考 (college entrance exam), 喜欢 (like), 事 (things), 专注 (focus), 挖 (dig) |
| 5 | 我航 (Wohang), 报国 (serve the nation), 强军 (strengthen the military), 图片 (image), 晚安 (good night), 早安 (good morning), 早安 (good morning) |
| 6 | 春暖 (warm spring), 花开 (flowers bloom), 路上 (on the road), 新 (new) |
| 7 | 前行 (move forward), 勇敢 (be brave), 远方 (the distance), 希望 (hope), 到达 (arrive) |
